# Preparing Medical Specialists to Practice Genomic Medicine: Education an Essential Part of a Broader Strategy

**DOI:** 10.3389/fgene.2019.00789

**Published:** 2019-09-11

**Authors:** Erin Crellin, Belinda McClaren, Amy Nisselle, Stephanie Best, Clara Gaff, Sylvia Metcalfe

**Affiliations:** ^1^Australian Genomics Health Alliance, Melbourne, VIC, Australia; ^2^Genomics in Society, Murdoch Children’s Research Institute, Melbourne, VIC, Australia; ^3^Department of Paediatrics, The University of Melbourne, Melbourne, VIC, Australia; ^4^Australian Institute of Health Innovation, Macquarie University, Sydney, NSW, Australia

**Keywords:** medical specialist, workforce, genomic medicine, preparedness, theory, genomic education, review

## Abstract

Developing a competent workforce will be crucial to realizing the promise of genomic medicine. The preparedness of medical specialists without specific genetic qualifications to play a role in this workforce has long been questioned, prompting widespread calls for education across the spectrum of medical training. Adult learning theory indicates that for education to be effective, a perceived need to learn must first be established. Medical specialists have to perceive genomic medicine as relevant to their clinical practice. Here, we review what is currently known about medical specialists’ perceptions of genomics, compare these findings to those from the genetics era, and identify areas for future research. Previous studies reveal that medical specialists’ views on the clinical utility of genomic medicine are mixed and are often tempered by several concerns. Specialists generally perceive their confidence and understanding to be lacking; subsequently, they welcome additional educational support, although specific needs are rarely detailed. Similar findings from the genetics era suggest that these challenges are not necessarily new but on a different scale and relevant to more specialties as genomic applications expand. While existing strategies developed for genetic education and training may be suitable for genomic education and training, investigating the educational needs of a wider range of specialties is critically necessary to determine if tailored approaches are needed and, if so, to facilitate these. Other interventions are also required to address some of the additional challenges identified in this review, and we encourage readers to see education as part of a broader implementation strategy.

## Introduction

Genomic medicine (i.e., the use of genomic information to guide diagnostic and treatment decisions) promises to transform the way medicine is practiced (Collins and McKusick, 2001; Williams, 2019). However, numerous challenges must be overcome for this promise (illustrated in **Table 1**) to be realized, including developing a competent workforce (Manolio et al., 2013; Bowdin et al., 2016). Medical specialists without specific genetic qualifications (defined herein as doctors specialized in a field other than general/family practice or clinical/medical genetics) will be key players in this workforce; however, their preparedness to practice genomic medicine has long been questioned (Guttmacher et al., 2001; Slade and Burton, 2016). It is widely feared that limited medical specialist knowledge and/or skills may see genomic tests misused or not used at all, to the detriment of patient care (Passamani, 2013; Korf et al., 2014; Burton et al., 2017). Consequently, there have been calls for educational efforts across the spectrum of medical training [i.e., from medical school and specialty training to continuing medical education (CME) (Guttmacher et al., 2007; McGrath and Ghersi, 2016)], with upskilling practicing medical specialists *via* CME the focus of this review.

**Table 1:** Case study illustrating the promise of genomic medicine, derived from existing literature (Notarangelo and Fleisher, 2017; Stray-Pedersen et al., 2017).

**Case** Rose is in her late teens and is continually in and out of hospital; she has suffered from serious, recurrent lung infections and autoimmune disease since childhood. Rose is suspected to have a primary immunodeficiency (PID), but a precise diagnosis remains elusive, despite repeated cellular and genetic testing.
**Utility of Genomic Testing** Rose’s immunologist recently heard about the diagnostic utility of genome sequencing, considers Rose a suitable candidate and hopes sequencing numerous genes in parallel may provide Rose with a more specific diagnosis and, potentially, help inform her treatment.
**Outcome** Genomic testing pinpoints the genetic variant responsible for Rose’s PID. This variant leads to overactivation of a protein that drives lymphocyte proliferation. Targeting this overactivated cell pathway with a readily available immunosuppressant is known to alleviate the severity of patients’ disease. An immunosuppressant paradoxically helps treat an immunodeficiency. Without a genomic diagnosis, Rose’s immunologist would never have thought of prescribing such a drug.

### Towards Effective Genomic Education

In response to the broader call for increased genomic education for medical specialists, the concept of what constituted a “prepared” medical specialist began to be considered. Vassy et al. (2015), drawing upon the competencies developed by Korf et al. (2014), proposed physicians would be sufficiently prepared if they had the knowledge and skills required to navigate genomic medicine and incorporate it into patient care. They stressed that as genomic practices are likely to be diverse, the nature of the knowledge and skills required will likely vary for different medical specialists. Yet, specific details as to how this might be successfully achieved were lacking from these early claims.

Here, we build upon this work and define preparedness as having the competence (knowledge, skills, and attitudes) and confidence to practice genomic medicine (whether it be identifying and referring suitable patients, or ordering and interpreting genomic tests) and propose that it could be achieved with greater efficacy and efficiency if CME approaches were grounded in adult learning theory.

#### Adult Learning Theory

According to adult learning theory, education of adults is most effective when they recognize a need to learn (i.e., when they are interested) and when education is tailored to the needs they self-identify, which arise from their work setting (Grant, 2002; Knowles et al., 2015). Problem-centered learning is preferred, as adults are keen to acquire knowledge and skills that are immediately applicable to real-life settings.

It is critical to emphasize that one cannot PRESUME a need to learn exists (Metcalfe et al., 2008) or that medical specialists will even be receptive to genomic medicine. After all, advances in genomics are not occurring in isolation; medical specialists have numerous competing learning demands and areas of interest (Feero et al., 2014). Investigating medical specialists’ willingness to learn and potential educational needs is an essential first step and, as Reed et al. (2016) and others (Gaff et al., 2007; Houwink et al., 2011; Houwink et al., 2014) show, facilitates the design and delivery of effective educational interventions.

Here, we review what is currently known about medical specialists’ perceptions of genomic medicine. Do specialists see a role for genomic medicine in their specialty, now or in the future? Would they feel confident using genomic tests? What do they know or think they should know about genomic testing and its use in clinical practice?

## Review Methodology

Initial searches of empirical literature on medical specialists’ perceptions of genomics yielded limited results. As we believed that useful insights could be gained from medical specialists’ earlier experiences with genetics, our literature search was subsequently broadened to include perceptions of genetic tests, too. Here, we define a genetic test as that which analyzes a single gene one at a time and a genomic test as that which analyses scores of (or all) genes simultaneously [see Brittain et al. (2017) for an overview of gene panels, whole exome sequencing (WES), and whole genome sequencing (WGS)]. Searches were conducted in MEDLINE, Embase, and PubMed using the search strategy detailed in the **Supplementary Material**, with articles focused on both germline and somatic testing examined.

## Genetics, Genomics, and Medical Specialists: A Complex Relationship

### Perceived Utility and Concerns

Views regarding the perceived relevance of genetics to conditions seen in clinical practice and utility of genetic testing varied across and within the specialties studied in the literature (Wilkins-Haug et al., 2000a; Hoop et al., 2008a; Hoop et al., 2008b; Harris et al., 2013; Myers et al., 2016; Amara et al., 2018; Diamonstein et al., 2018; Loss et al., 2018). For example, genetics was considered highly relevant and useful in obstetrics and pediatrics (Diamonstein et al., 2018) but less so in psychiatry (Hoop et al., 2008b) and general internal medicine (Diamonstein et al., 2018). Perceived utility is known to influence test use (Sanson-Fisher, 2004), exemplified by oncologists’ rapid embrace of KRAS^1^ genetic testing for metastatic colorectal cancer when they were convinced that such testing would usefully inform treatment decisions (Harris et al., 2013).

Although the value of genetic testing was often recognized, a number of concerns, primarily relating to test access and implications for patients, were often raised across studies (Freedman et al., 2003; Finn et al., 2005; Harris et al., 2013; Salm et al., 2014; Myers et al., 2016). Perceptions of genomics, which largely emanate from the oncology field to date, are proving to be similarly mixed, with perceived benefits often tempered by a host of concerns, some old, some new.

Some oncologists (Gray et al., 2014; Chow-White et al., 2017; Johnson et al., 2017), pediatric neurologists (Jaitovich Groisman et al., 2017), and neonatologists (Knapp et al., 2019) believed that genomic tests would be useful for facilitating diagnoses and family planning, guiding treatment selection, or aiding disease surveillance. Yet, across studies, many specialists questioned the current utility of genomic testing (Miller et al., 2014; Chow-White et al., 2017; Deininger et al., 2019; Knapp et al., 2019), with few treatments available and genomic information yet to be fully deciphered.

Of the limited studies conducted to date, concerns raised included genomic test access and cost (Helman et al., 2016; Chow-White et al., 2017; Jaitovich Groisman et al., 2017), lack of evidence and clinical guidelines (Bonter et al., 2011; Stanek et al., 2012; Amara et al., 2018), and the potential for genomic tests to cause psychological harm or impede insurance access (Johnson et al., 2017; Deininger et al., 2019; Knapp et al., 2019). These concerns linger from the genetics era, with additional worries arising from the complexity, volume, and uncertain nature of the data generated (Miller et al., 2014; Christensen et al., 2016; Gray et al., 2016; Knapp et al., 2019). For instance, some oncologists (Gray et al., 2016; Weipert et al., 2018) and cardiologists (Christensen et al., 2016) participating in various genomics studies were worried about being burdened with the responsibility of disclosing additional findings (e.g., cancer predispositions or conditions that lay outside their specialty). Other oncologists (Miller et al., 2014; McCullough et al., 2016) were troubled by the potential for additional findings to cause undue worry or distract patients or parents from their/their child’s primary condition. Yet, despite holding numerous concerns, specialists often saw the infiltration of genomics into medicine as inevitable (Selkirk et al., 2013; Chow-White et al., 2017; Jaitovich Groisman et al., 2017), an inevitability, as indicated in the section that follows, for which few felt prepared.

### Understanding and Confidence

Medical specialists’ perceived or actual knowledge of genetic concepts, conditions, and/or testing have long been shown to be highly variable and frequently poor (Hofman et al., 1993; Hunter et al., 1998; van Langen et al., 2003; Baars et al., 2005; Hoop et al., 2008b; Nippert et al., 2011; Klitzman et al., 2013). Canadian specialists surveyed by Hunter et al. (1998), for instance, had poor knowledge of the availability of genetic tests for specific conditions, with further studies suggesting that knowledge levels have not improved since. For example, the majority of European primary care specialists (pediatricians and obstetrician–gynecologists) surveyed by Nippert et al. (2011) expressed limited confidence in their ability to identify/explain inheritance patterns and perform other such tasks, and most US specialists surveyed by Klitzman et al. (2013) perceived their genetic knowledge to be very/somewhat poor. That said, genetic knowledge often varied by specialty. Specialties and subspecialties (for example, cardiologists subspecialized in cardiogenetics) with greater genetics exposure often had higher perceived or actual knowledge of genetic concepts, conditions, and/or testing (Hofman et al., 1993; Pichert et al., 2003; van Langen et al., 2003; Baars et al., 2005; Nippert et al., 2011). These studies imply that the impetus to know about genetics is greatest when it is perceived to be directly relevant to one’s clinical practice, in line with adult learning theory.

Comfort to discuss or use genetics in practice, while often low (Klitzman et al., 2013), also differed across specialties, reflected in the various roles that specialists were willing to assume. Neurologists, for instance, appeared to be more comfortable ordering genetic tests and interpreting and discussing test results compared with psychiatrists (Finn et al., 2005; Salm et al., 2014; Zhou et al., 2014; Dominguez-Carral et al., 2017). Self-confidence was often a product of genetics experience (with neurologists in the preceding example having cause to use genetic tests more frequently than psychiatrists) and a predictor of future test use (Freedman et al., 2003; Salm et al., 2014). Moreover, those who had received some genetic education were often more confident and knowledgeable and used genetics more frequently (Hofman et al., 1993; Wilkins-Haug et al., 2000b; Hoop et al., 2008b; Nippert et al., 2011), supporting a role for education in facilitating competent practice.

A similar lack of preparedness is emerging from the genomics literature, with specialists mostly expressing low confidence in their understanding of, and ability to use, somatic or germline genomic tests (Bonter et al., 2011; Selkirk et al., 2013; Gray et al., 2014; Amara et al., 2018; Deininger et al., 2019; Knapp et al., 2019) but self-reporting familiarity with basic genetic concepts (Stanek et al., 2012; Chow-White et al., 2017). Knowledge and confidence have often been shown to be highest among oncologists compared with other specialties (Bonter et al., 2011; Stanek et al., 2012); however, confidence levels are even relatively low among this experienced group of genetic/genomic test users, particularly with regards to germline results (Chow-White et al., 2017; Johnson et al., 2017; Weipert et al., 2018).

Findings from somatic and/or germline studies with oncologists (Chow-White et al., 2017; Johnson et al., 2017; Weipert et al., 2018) and neonatologists (Knapp et al., 2019) indicate comprehending and communicating genomic information, with colleagues or patients, will be challenging for most. However, there is some evidence, albeit from a small qualitative study of pediatric oncologists involved with tumor genomics (McCullough et al., 2016), to suggest that some individuals do not consider genomic information as any more complex or daunting to communicate than the tasks they currently perform.

Given specialists’ perceived lack of preparedness to practice genomic medicine, it is unsurprising that many strongly supported additional education, training, and resources, such as clinical guidelines (Bonter et al., 2011; Selkirk et al., 2013; Chow-White et al., 2017; Jaitovich Groisman et al., 2017; Weipert et al., 2018; Deininger et al., 2019). Yet, few studies have explored specialists’ preferences in any great depth (Selkirk et al., 2013; Weipert et al., 2018; Deininger et al., 2019). The survey of oncologists involved with a tumor genomics study by Chow-White et al. (2017) suggests that specialists are keen to learn about the practicalities of genomic testing and that participating in genomics research is a useful means of gaining knowledge and skills, but further evidence is severely lacking.

Education is likely to work best when key conditions are met; for instance, clinical utility is recognized. Neurologists, in the study by Jaitovich Groisman et al. (2017), who saw a use for genome sequencing in their future practices were far more supportive of education compared with those who did not. Clearly, exploring medical specialists’ perceptions of genomic medicine is a useful starting point for gauging interest in, and need for, educational support.

Self-confidence and perceived competence remain strong predictors of future (somatic or germline) test use (Gray et al., 2014; Johnson et al., 2017). A qualitative study by Weipert et al. (2018) also suggests that these constructs are a product of one’s work setting. Several oncologists in their study felt that community-based practitioners had less exposure to genomics than their counterparts working in academic/tertiary settings and therefore would be less competent ordering and interpreting genomic tests. This perception is yet to be verified, though. Confidence and perceived competence additionally appear to be products of genomic education and experience (Bonter et al., 2011; Stanek et al., 2012; Selkirk et al., 2013; Amara et al., 2018), once again supporting a role for education in facilitating competent practice.

## Role of Education in Preparing Medical Specialists to Practice Genomic Medicine

This review suggests a range of factors (e.g., perceived utility and consequences, confidence, experience level, education, and resources available) are likely to influence medical specialists’ preparedness to practice genomic medicine, echoing findings from a systematic review by Paul et al. (2018) of factors influencing medical specialists’ use of genetic tests. In light of this, it is worthwhile reflecting on the role education will play in mainstreaming genomic medicine. To do so, we need to take a step back and see where education fits within a broader genomic medicine implementation strategy.

Implementation science is a field that uses a range of behavior change theories to systematically study ways of getting evidence into practice (Michie et al., 2005; Bauer et al., 2015). As part of a learning healthcare system, focused on continual improvement and where collaboration among diverse stakeholders including clinicians is critical, implementation science can provide the mechanism for considering future implementation strategies (Chambers et al., 2016; Gaff et al., 2017). Different theories can be used to study potential barriers and facilitators, guide the selection and implementation of suitable behavior change interventions, and subsequently evaluate intervention efficacy (Lynch et al., 2018). Theories are used to create generalizable results and because it is widely recognized that theory-driven approaches are more likely to work (Bauer et al., 2015).

The Capability, Opportunity, and Motivation Model of Behavior (COM-B model) is a theory commonly used to identify barriers and facilitators to the adoption of new practices like genomic medicine and to aid the selection of behavior change interventions, for example, educational supports (Michie et al., 2011; McDonagh et al., 2018). According to this model, three interacting constructs result in a behavior (e.g., referring patients/ordering genomic tests to guide diagnostic and treatment decisions): capability, the requisite knowledge and skills; opportunity, the support of a well-resourced environment and one’s peers; and motivation, the self-belief that one is capable of performing a given task and the practice will lead to positive, not negative, outcomes. These constructs, illustrated in the context of genomic medicine, are further detailed in **Table 2**.

**Table 2:** The Capability, Opportunity, Motivation Behavior (COM-B) model adapted from Michie et al. (2011) to apply to genomic medicine. Three intersecting constructs will likely determine the successful use of genomic medicine in medical specialist practice. The capability and motivation constructs can be amenable to education and training.

Construct	Illustrated in the Context of Genomic Medicine
**Capability**	The knowledge and skills to know: when testing could be useful; how to appropriately refer/order testing; and how to interpret and communicate test results, plus understand the implications for patients and families.
**Opportunity**	The ability to: physically access and use genomic testing (resources like adequate time and funding must be available); and work in an environment where genomic testing is used and where peers can be lent upon for support.
**Motivation**	The belief that: genomic testing will be clinically useful and lead to positive, not negative, consequences; genomic testing is compatible with existing professional roles; and one can competently use genomic information to guide patient care.

As various aspects of these three constructs can enhance or impede behavior change (Michie et al., 2011), a suite of interventions will likely be needed to see genomic medicine successfully integrated into routine practice. The capability and motivation constructs can be amenable to education and training, supported by evidence from systematic reviews (Grol and Grimshaw, 2003; Paneque et al., 2016; Talwar et al., 2017), which consistently show that education can help improve competence (knowledge, skills, and attitudes) and confidence. However, behavior change with education alone is rare. This may be because: a) long-term outcomes are difficult to measure (although attempts to measure these outcomes should be considered prior to developing educational interventions; Talwar et al., 2017) and b) other interventions need to be delivered alongside education for education to have additional impact (Grol and Grimshaw, 2003). CME in genomic medicine is likely to be most effective as an essential part of a broader implementation strategy.

Since multidisciplinary teamwork is known to be critical for successful implementation (Chambers et al., 2016), upskilling other health professions who work alongside medical specialists and providing interdisciplinary education to those working in the same clinical setting, previously identified as being relevant to genetics (Gaff et al., 2008), is additionally and importantly needed.

## Summary and Conclusions

Findings from the very limited empirical studies conducted to date (largely in the field of oncology) suggest that medical specialists’ perceptions of genomic medicine are likely to be complex. Mixed views on the clinical utility of genomic medicine currently exist, with perceived benefits frequently tempered by several concerns. At the same time, specialists generally consider the arrival of genomic medicine inevitable. Most do not feel prepared for this inevitability and perceive a lack of understanding and confidence. While little evidence exists, there is indication that CME in genomic medicine is likely to be broadly welcomed.

Similar findings from the genetics era suggest that these challenges are not necessarily new in the genomics era but occur on a larger scale and are likely to be relevant to more specialties as genomic applications expand across medicine (Burton et al., 2017; Knapp et al., 2019). Informing medical specialists that genomics is, in many ways, a continuation of genetics may be reassuring to those daunted by the impending arrival of genomic medicine. It also suggests that existing strategies for genetic education and training may be transferable to genomic education and training. Given the limited resources available for genomic education, repurposing and sharing educational materials, where possible, through online repositories will be important (Nisselle, submitted). Equally important will be improving the quality of evaluation approaches, noting that while existing educational strategies may be transferable across different settings, evaluation of these strategies will likely need to be different (Talwar et al., 2017). Current efforts often lack methodological rigor, are infrequently guided by theory, and rarely include follow-up data to determine long-term impact. The COM-B model introduced in this review could be one theory used to guide evaluation approaches.

To test the hypothesis that existing educational strategies may be transferable, a perceived need must be confirmed. Detailed explorations of educational needs should be undertaken, in a wider range of medical specialties and more diverse settings (most studies reviewed arose from academic/tertiary hospitals; the needs of community-based practitioners are largely unknown). Findings from the genetics era revealed that the perceived relevance of genetics varied across specialties. Whether this remains the case in the genomics era is unknown and worth investigating.

We are investigating the perceptions, experiences, and education and training needs of varied health professionals (including medical specialists) in the Workforce & Education program of the Australian Genomics Health Alliance (Stark et al., 2019). Guided by the principles of adult learning theory, which have previously informed health professional genetic/genomic needs assessments (Gaff et al., 2007; Metcalfe et al., 2008; Reed et al., 2016), we seek to investigate the perceived relevance of genomic medicine to clinical practice and document education and training needs, should they exist. Establishing this evidence base will be critical to facilitate the implementation of tailored educational supports.

## Author Contributions

EC, CG, SM, AN, and BM conceived the idea for the manuscript, and EC conducted the literature searches, reviewed the literature, and drafted and revised the manuscript. CG, SM, AN, and BM provided intellectual input throughout and revised drafts. SB provided substantial input into the implementation science component and revised drafts. All authors approved the final version and agree to be accountable for all aspects of the work.

## Funding

This work was supported by the Victorian Government’s Operational Infrastructure Support Program and a grant from the Australian National Health & Medical Research Council (GNT1113531); the contents are solely the responsibility of the individual authors and do not reflect the views of the NHMRC. EC was supported by The University of Melbourne Helen R. Freeman Scholarship.

## Conflict of Interest Statement

The authors declare that the research was conducted in the absence of any commercial or financial relationships that could be construed as a potential conflict of interest.

## Footnotes

1 Kirsten ras (KRAS) tumor mutation.
