# Supplementary material for: Preparing Medical Specialists to Practice Genomic Medicine: Education an Essential Part of a Broader Strategy
Source: Front Genet. 2019 Sep 11;10:789. doi: 10.3389/fgene.2019.00789 (PMC6749815; doi:10.3389/fgene.2019.00789)
Supplement: Supplementary file 1 [file DataSheet_1.docx]

Crellin et al. (2019) Preparedness to Practice Genomic Medicine

Supplementary Material

**1. Literature Search Strategy**

**1.1. Search Terms**

Literature searches were conducted in MEDLINE, Embase and PubMed using variations of the following string of terms: (“genomics” OR “genome” OR “next-generation sequencing” OR “whole exome sequencing” OR “whole genome sequencing” OR “precision medicine” OR “pharmacogenomics” OR “genetic services” OR “medical genetics” OR “genetic predisposition”) AND (“physicians” NOT “family physicians” OR “general practitioners”) AND (“attitudes” OR “practice patterns” OR “competence” OR “clinical practice” OR “health care delivery” OR “self-efficacy” OR “professional development” OR “medical education” OR “knowledge”). Additional articles were retrieved by reviewing reference lists.

**1.2. Exclusion and Inclusion Criteria**

Articles were included if they were primary studies focused on medical specialists’ perceptions and knowledge of germline or somatic genetic/genomic testing for a clinical indication (e.g., predisposition testing in the presence of a strong family history), published in English between 1990 (the year the Human Genome Program commenced) and 2019.

Articles were excluded if they focused on population-based screening, carrier or direct-to-consumer (online DNA) testing, non-invasive prenatal testing (NIPT) or preimplantation genetic diagnosis (PGD). Articles were also excluded if they focused on the evaluation of an educational intervention.

Additionally, articles were excluded if they solely featured general practitioners/family physicians or clinical/medical geneticists; these professions were not the focus of this review, as indicated in the main paper. However, due to the paucity of the available literature, articles containing a mixture of physicians were examined if findings were stratified by specialty (e.g., GPs versus medical specialists) or, if results were not stratified, medical specialists without specific genetics qualifications constituted the majority (i.e. ≥ 50%) of the study sample.
